# An Atypical Case of Sjögren's Syndrome: A Surprise Diagnosis

**DOI:** 10.7759/cureus.84807

**Published:** 2025-05-25

**Authors:** Syed Hassan, Sara Samreen, Faiz Fathima Shaik, Syeda Maariyah Hashmi, Abdul Shafi

**Affiliations:** 1 Department of Medicine, Deccan College of Medical Sciences, Hyderabad, IND

**Keywords:** autoimmune disorder, autoimmune rheumatologic diseases, distal renal tubular acidosis, hypokalemia, hypokalemic periodic paralysis, renal biopsy, sjögren’s syndrome, tubulointerstitial nephritis

## Abstract

Distal renal tubular acidosis (RTA) presenting as hypokalemia, metabolic acidosis, and hypokalemic periodic paralysis is a common manifestation of tubulointerstitial nephritis (TIN) in Sjögren’s syndrome. Sjögren’s syndrome is a chronic, progressive, systemic autoimmune inflammatory disease characterized by lymphocytic infiltration of exocrine glands, which results in sicca syndrome, i.e., xerostomia and keratoconjunctivitis sicca. Some patients can express sicca symptoms with other autoimmune diseases. It is mostly seen in females, and middle-aged women are more likely to be affected. Some patients can present with extra-glandular (systemic) manifestations, of which renal involvement is the most common. Renal involvement in the form of TIN is more common compared to glomerular involvement. We report a case of a 37-year-old female presenting with hypokalemic periodic paralysis, later diagnosed as distal RTA secondary to Sjögren’s syndrome, which requires high clinical suspicion based on hypokalemia refractory to treatment. Further evaluation is necessary to confirm the diagnosis.

## Introduction

Sjögren's syndrome is a chronic autoimmune inflammatory disease of the exocrine glands, predominantly the salivary and lacrimal glands, leading to sicca symptoms characterized by keratoconjunctivitis sicca and xerostomia. It can be primary (occurring in isolation) or secondary when associated with other autoimmune connective tissue diseases such as rheumatoid arthritis, systemic lupus erythematosus, and scleroderma [1]. It is known to affect middle-aged women with an average female-to-male ratio of 9:1, usually being diagnosed in the fifth decade of life, with a mean age of onset ranging from 51.6 (±13.8) to 62 (±13) years [2].

Apart from classic sicca symptoms, it is also known to involve other organ systems. Extra-glandular manifestations (EGMs) of Sjögren's syndrome are complex and are divided into non-visceral symptoms, including musculoskeletal and cutaneous manifestations, and visceral symptoms, including renal, neurological, hematological, gastrointestinal, cardiovascular, and pulmonary manifestations [3]. Renal involvement in Sjögren's syndrome is rarely defined. The involvement of the kidneys is noted only in 9% of cases with primary Sjögren's syndrome [4]. It is divided into three types: (1) tubulointerstitial nephritis (TIN), characterized by peritubular infiltration of lymphocytes; (2) glomerulonephritis (GN), associated with deposition of immune complexes; and (3) disorders associated with the presence of specific autoantibodies [5]. TIN is found to be the most common histological pattern. Distal renal tubular acidosis (dRTA) is the most common presentation in these patients. Hypokalemia and metabolic acidosis in dRTA cause muscle weakness and periodic paralysis [6]. However, severe hypokalemic periodic paralysis due to dRTA in Sjögren's syndrome is very rare. Here, we report a case of Sjögren's syndrome that presented with hypokalemic periodic paralysis, which was found to be secondary to distal renal tubular acidosis as a consequence of TIN due to Sjögren's syndrome.

## Case presentation

A 37-year-old female with no known comorbidities came with complaints of recurrent, relapsing, and remitting patterns of motor weakness of all four limbs for the past three years, with no history of sensory or autonomic dysfunction. She was previously treated as hypokalemic periodic paralysis of unknown cause and responded well to potassium (K+) replacement therapy. On further enquiring, she reported the presence of generalized body pains, dryness of eyes, small joint pains of both the upper limbs, and backache for the past two years.

During the general physical examination, signs of dehydration were noted, including dryness of the mouth, and no dental caries were observed. No obvious parotid gland, submandibular gland, or lacrimal gland enlargement was noted. The thyroid gland was normal in size and non-tender. No lymphadenopathy was noted. Mild joint tenderness of the bilateral proximal interphalangeal joints and metacarpophalangeal joints was observed. Skin examination revealed dryness of the skin with no rash. Her vitals were normal. The cardiovascular, gastrointestinal, and respiratory systems showed no abnormality. Neurological examination revealed that the patient had flaccid weakness of both upper and lower limbs with power of 1/5, no cranial nerve involvement, and no sensory and autonomic dysfunction.

On laboratory investigations, she was found to have iron deficiency anemia and anemia of chronic disease, with hemoglobin at 9.2 gm/dl, mean corpuscular volume of 89 fl, mean corpuscular hemoglobin at 26.6 pg, mean corpuscular hemoglobin concentration of 29.9 g/dl, serum iron at 28 mcg/dl, serum ferritin at 852 ng/ml, serum total iron-binding capacity (TIBC) of 320 mcg/dl, and transferrin saturation of 12%.

Serum electrolytes test showed serum sodium at 141 mmol/l, serum potassium at 2.6 mmol/l, serum chloride at 108 mmol/l, serum bicarbonate at 20.2, normal anion gap (12.8, normal is 8-16), and serum magnesium at 2.2 mg/dl (1.8-2.8 mg/dl). Blood urea was 32 mg/dl, and serum creatinine was 0.9 mg/dl. Markedly decreased potassium levels led us to a workup of hypokalemia.

Urine biochemistry revealed urine pH of 7.8 (alkaline), 24-hour urinary potassium of 16 mmol/day, spot urine potassium creatinine ratio of 15 mmol/gm, transtubular potassium gradient of >5, urine bicarbonate at 5 mmol/L, chloride at 217 mmol/L, phosphorous at 1.2 mg/dl, urine creatinine at 15 mg/dl, urine protein-creatinine ratio of 1.15, and 24-hour urinary protein at 1527 mg/day (proteinuria), which in the setting of hypokalemia suggested renal potassium loss.

Arterial blood gas (ABG) analysis showed normal anion gap metabolic acidosis, with pH of 7.30, partial pressure of carbon dioxide (pCO2) of 24, partial pressure of oxygen (pO2) of 94, arterial bicarbonate (HCO3) of 20.2, and normal anion gap (12.2, normal is 8-16). Venous bicarbonate was 20.8 mEq/l. The urine anion gap was found to be 96, which, in the setting of low potassium and high urinary pH, led us to a diagnosis of type 1 renal tubular acidosis (RTA).

Serum 25-OH vitamin D level was 13.9 ng/ml (normal: >30 ng/ml), serum phosphorus was 1.2 mg/dl, and serum calcium was 7.7 mg/dl. Thyroid profile was normal, with thyroid-stimulating hormone (TSH) at 3.5 mIU/L, T3 at 1.2 ng/ml, and T4 at 7.2 mcg/dl. The renal function test was normal. The liver function test was also normal, except for albumin at 3.2 gm/dl (hypoalbuminemia). Rheumatoid factor was <8 IU/ml (negative), C-reactive protein was <6 mg/dl (negative), and erythrocyte sedimentation rate at first hour was 90 mm and at second hour was 125 mm. Viral markers (HIV, hepatitis B surface antigen, and hepatitis C virus) were negative (Table 1).

**Table 1:** Laboratory investigations. TIBC: total iron-binding capacity; TSH: thyroid-stimulating hormone; T3: triiodothyronine; T4: thyroxine; RA factor: rheumatoid factor; CRP: C-reactive protein; ESR: erythrocyte sedimentation rate; HIV: human immunodeficiency virus; HBsAg: hepatitis B surface antigen; HCV: hepatitis C virus; pCO2: partial pressure of carbon dioxide; pO2: partial pressure of oxygen; HCO3: bicarbonate.

Investigations	Result	Reference range
Hemoglobin	9.2 g/dl	12.0-15.8 g/dL
Mean corpuscular volume	89 fl	83-101 fl
Mean corpuscular hemoglobin	26.6 pg	27-32 pg
Mean corpuscular hemoglobin concentration	29.9 g/dl	32-36 g/dl
RBC count	3.4 million/cumm	Female: 3.8-5.0 million/cumm
WBC count	4600 cumm	4000-11000 cumm
Platelet count	2.6 lakhs/cumm	1.5-4.5 lakhs/cumm
Serum iron	28 mcg/dl	41-141 mcg/dL
Serum ferritin	852 ng/ml	10-150 ng/mL
Serum TIBC	320 mcg/dl	251-406 mcg/dL
Transferrin saturation	12%	16-35%
Serum Na^+^	141 mmol/l	136-145 mmol/L
Serum K^+^	2.6 mmol/l	3.5-5.0 mmol/L
Serum Cl^-^	108 mmol/l	95-105 mmol/L
Serum magnesium	2.2 mg/dl	1.6-2.3 mg/dL
Blood urea	32 mg/dl	10-45 mg/dl
Serum creatinine	0.9 mg/dl	0.6-1.5 mg/dl
Urinary potassium (24 hours)	16 mmol/day	<15 mmol/day
Spot urine K^+^	15 mmol/gm creatinine	<13 mmol/gm creatinine
Urine pH	7.8	5.5-7.5
Urine sodium	177 mmol/l	100-260 mmol/l
Urine potassium	136 mmol/l	25-125 mmol/l
Urine bicarbonate	5 mmol/L	<1-2 mmol/L
Urine protein	17 mg/dl	0-15 mg/dl
Urine chloride	217 mmol/L	110-250 mmol/L
Urine phosphorous	1.2 mg/dl	0.4-1.3 mg/dl
Urine creatinine	15 mg/dl	16-327 mg/dl
Urine protein creatinine ratio	1.15	Normal: <0.2; significant proteinuria: 0.2-3.5; nephrotic syndrome: >3.5
P24 hours urinary protein	1527 mg/day	<30 mg/day
pH	7.30	7.35-7.45
pCO_2_	24	32-45
pO_2_	94	83-108
Arterial HCO_3_	20.2	24-40
Venous bicarbonate	20.8 mEq/l	22-29 mEq/l
TSH	3.5 mIU/L	0.4-4.0 mIU/L
T3	1.2 ng/ml	0.8-2.0 ng/mL
T4	7.2 mcg/dl	5.0-12.0 mcg/dL
Serum 25-OH vitamin D levels	13.9 ng/ml	15-80 ng/mL
Serum PO4	1.2 mg/dl	2.5-4.3 mg/dL
Serum calcium	7.7 mg/dl	8.8-10.8 mg/dL
Serum albumin	3.2 g/dl	3.5-5 g/dL
RA factor	<8 IU/ml	<8 IU/ml - negative; ≥8 IU/ml - positive
CRP	<6 mg/dl	<6 mg/l - negative; ≥6 mg/l - positive
ESR	1st hour: 90 mm; 2nd hour: 125 mm	Females: 0-20 mm at 1^st^ hour
Viral markers (HIV, HbsAg, HCV)	Negative	Not applicable

**Figure 1:**
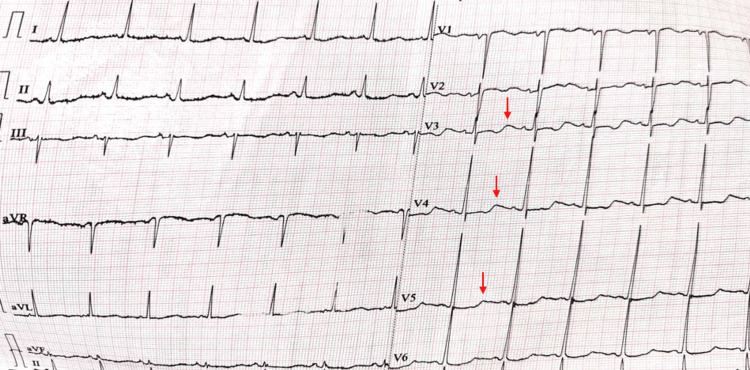
ECG in hypokalemia showing ST depressions and U waves. Red arrows showing U waves.

**Figure 2:**
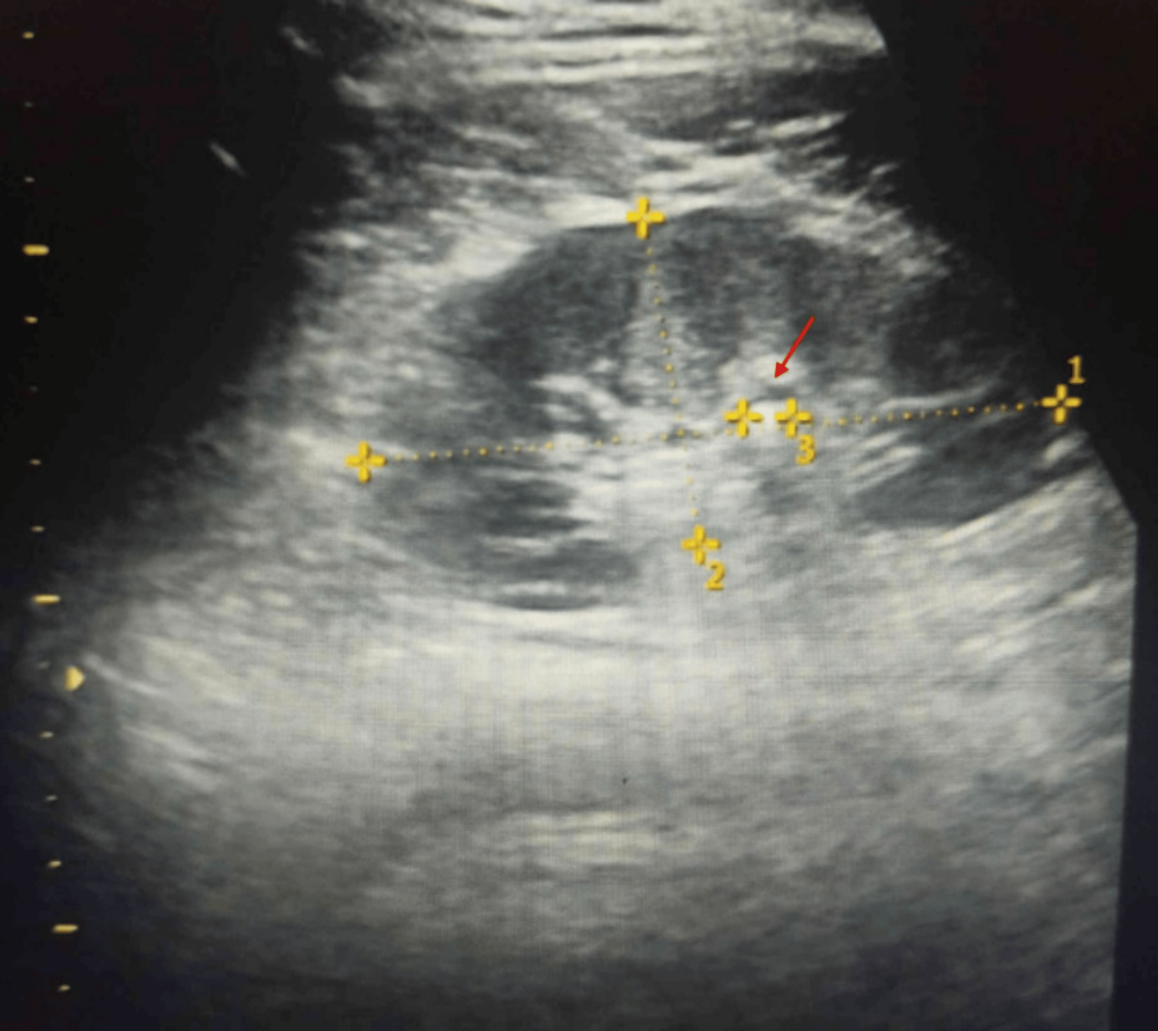
Ultrasound showing a 5 mm kidney stone. Red arrow showing a 5 mm stone.

ECG showed mild ST depression and U waves (Figure 1). Ultrasound examination showed normal kidneys with 5 mm left renal calculi in the lower pole (Figure 2). Diagnosis of distal RTA was considered, given hypokalemia (vs. hyperkalemia in type 4 RTA), with normal anion gap metabolic acidosis and alkaline urine (vs. acidic urine in type 2 RTA).

After working up the case for various causes of responsive hypokalemia and persistent proteinuria, with ABG showing acidosis, an antinuclear antibody (ANA) profile was ordered (ANA titers ≥ 640), which revealed anti-Ro52-3+ (strongly positive), and anti-SSB-3+ (strongly positive).

**Figure 3:**
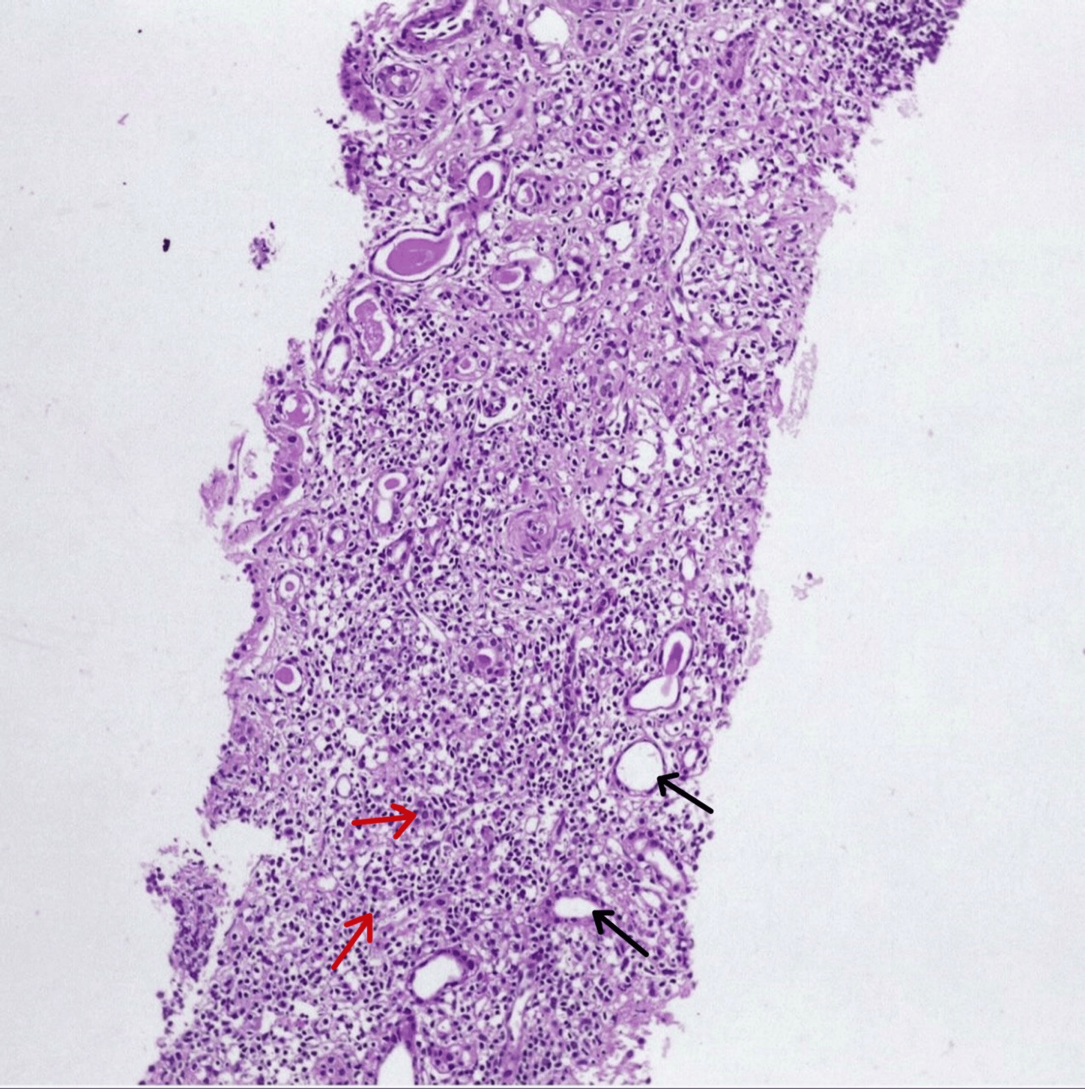
Renal biopsy showing tubulointerstitial nephritis. Hematoxylin & eosin stain showing infiltrate of mononuclear cells in the interstitium. Magnification is 40x. Black arrows pointing to tubules, and red arrows to inflammation in the interstitium.

**Figure 4:**
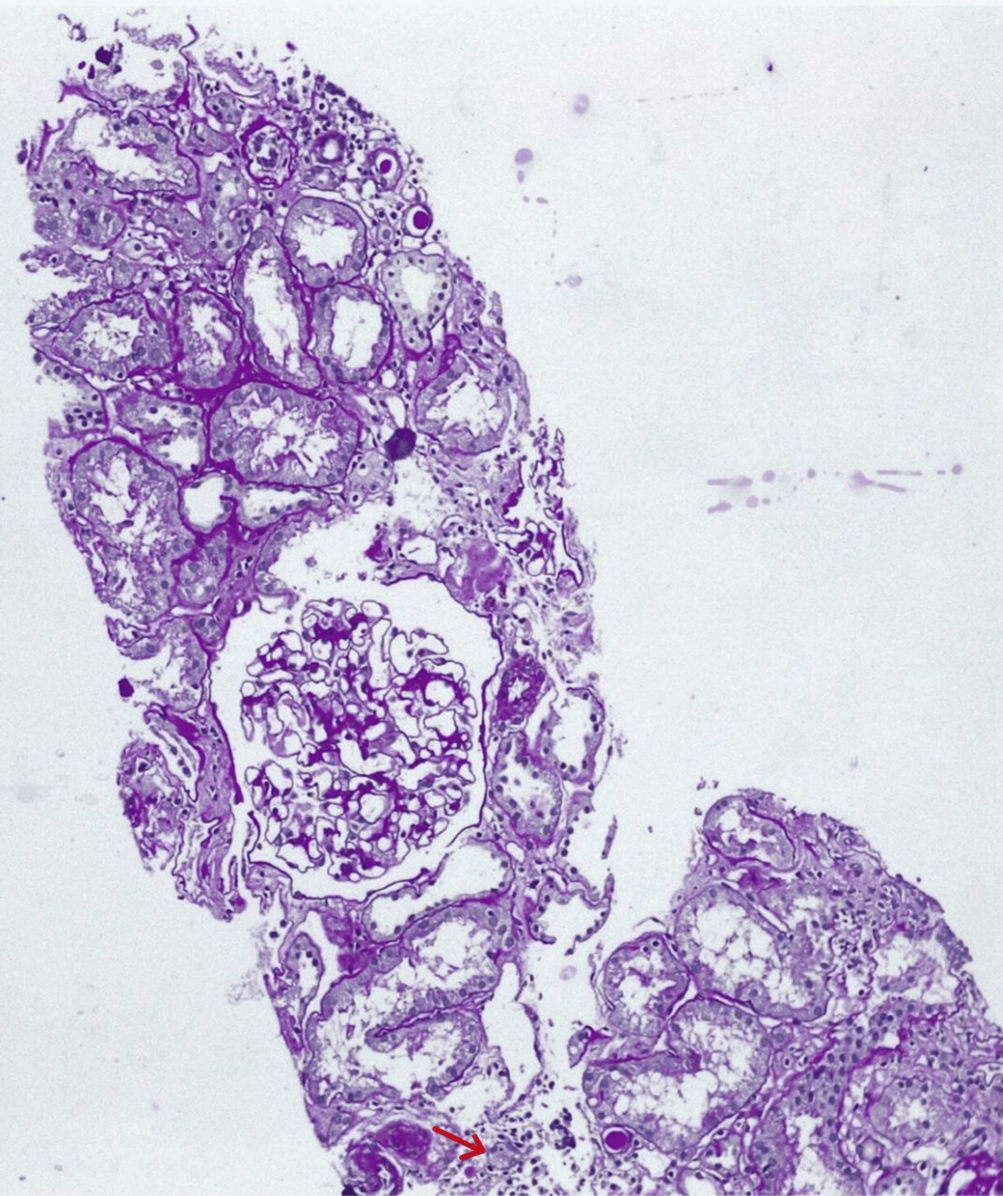
Renal biopsy showing tubulointerstitial nephritis. Periodic acid-Schiff (PAS) stain showing few tubules revealing flattened and denuded epithelium with focal mononuclear inflammation in the interstitium. Also, a few tubules showing hyaline casts. Magnification is 40x. Red arrow pointing to tubulitis.

Based on the clinical picture and ANA profile, a renal biopsy was done, which revealed features of TIN with interstitial inflammation and tubular injury and no significant immune deposits on immunofluorescence. This suggested the diagnosis of Sjögren's syndrome with tubulointerstitial disease (Figures 3, 4).

The patient was symptomatically treated for hypokalemia with potassium chloride. She was started on an oral low dose of prednisone 1 mg/kg once daily for four to six weeks, then tapered by 5-10 mg every one to two weeks over three months due to renal involvement (extra-glandular) along with hydroxychloroquine 200 mg once daily, and steroid-sparing drugs. Disease-modifying antirheumatic drug (DMARD), such as methotrexate 10 mg, was started once weekly to prevent long-term usage of steroids and their complications. Sodium bicarbonate was started at 3-4 mEq/kg/day. Artificial eye drops (hydroxypropyl methylcellulose) were used for ocular symptoms. Plenty of oral fluids were advised. The patient was instructed to refrain from using diuretics, tricyclic antidepressants (TCA), and antihistamines. The patient was also told about the high risk of developing lymphoma in the future.

The patient responded well to the treatment and showed significant improvement in her symptoms, with her motor strength being 5/5, and she was able to walk home. Labs were repeated, and potassium levels increased to 3.8 meq/l, which indicated an overall favorable prognosis. The patient was scheduled for a follow-up after three to four weeks, with lab investigations of serum potassium, urine potassium, and 24-hour urine protein to check for her treatment progress.

## Discussion

Primary Sjögren's syndrome is a systemic autoimmune disorder that is characterized by focal infiltration of lymphocytes in the exocrine glands, causing symptoms like dry eyes and dry mouth. However, glandular features similar to this can also appear as a late complication in patients having other rheumatic disorders such as systemic lupus erythematosus (SLE), rheumatoid arthritis (RA), and scleroderma (known as secondary Sjögren's syndrome). In approximately 70% of patients with primary Sjögren’s syndrome, anti-Ro and anti-La antibodies are found, usually with ANA positivity [7]. The underlying mechanism is an abnormal response of B and T cells to the autoantigens Ro/SSA and La/SSB, which destroy the exocrine gland epithelium [8]. The prevalence of Sjögren's syndrome is worldwide, with more cases being reported in adults than children, and no racial or geographic bias in incidence. The disorder has a marked predilection for females. It can affect any age group, but usually, symptoms appear in middle age, i.e., between the ages of 45 and 55 years [9].

Clinicians often find it difficult to diagnose Sjögren's syndrome as it exhibits a broad spectrum of clinical manifestations, ranging from mild exocrine symptoms of dry eyes, dry mouth, and myalgias to severe systemic disease leading to potentially life-threatening complications [10]. Thus, the disease usually goes undiagnosed when the patient’s initial presentation differs from the commonly encountered exocrine symptoms of dry eyes and mouth. This is due to the general emphasis on ocular and oral findings as diagnostic criteria. The American-European Consensus Group classification criteria require the presence of four of six criteria for the diagnosis of Sjögren's, which include (a) ocular or oral symptoms, (b) objective ocular or oral signs, (c) the presence of autoantibodies, and (d) histopathology from lip biopsy. In our patient, we found three out of the six criteria (dry eyes and mouth, and positive anti-SS-A and anti-SS-B antibodies), suggesting a possible diagnosis of primary Sjögren’s. Renal biopsy findings are not a part of the diagnostic criteria for Sjögren’s, but they can aid in diagnosis [11]. Renal involvement in primary Sjögren’s syndrome (pSS) was first documented in the 1960s, describing typical tubular defects [12]. The kidney is known to be the most affected non-exocrine organ, with the prevalence ranging between 2% and 67%. Interstitial nephritis is the most common type, followed by dRTA, nephrogenic diabetes insipidus, and glomerular diseases, which commonly include membranoproliferative glomerulonephritis (MPGN) and membranous nephropathy (MN) [13]. Interstitial nephritis (IN) evolves slowly, causing low-grade inflammation and occurs before or near the onset of typical sicca symptoms. Cryoglobulinemic glomerulonephritis in Sjögren's syndrome results from immune complex deposition. GN has an abrupt onset than IN but occurs late in the course of Sjogren's disease as nephritic syndrome with impaired renal function [14].

**Figure 5:**
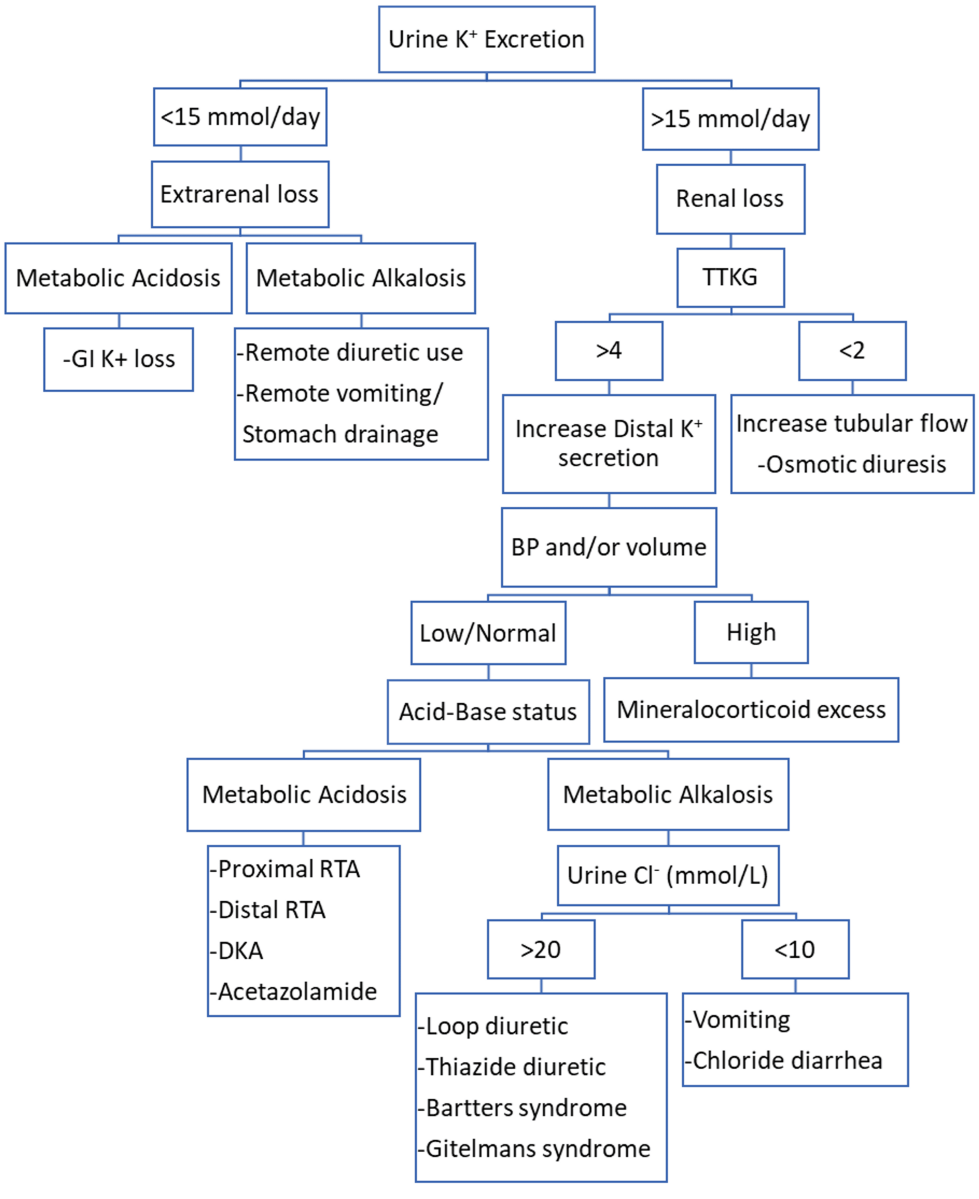
Approach to hypokalemia. TTKG: transtubular K+ concentration gradient; GI: gastrointestinal; RTA: renal tubular acidosis; DKA: diabetic ketoacidosis. Source: [15]. Figure credits: Sara Samreen and Syed Hassan.

Clinically, the most common electrolyte imbalance observed is hypokalemia. Figure 5 shows an approach to hypokalemia [15]. There are many causes of hypokalemia, which can be classified into three types: excessive excretion, insufficient intake of potassium, or changes in the distribution of intracellular and extracellular potassium levels. Endocrine diseases with renal potassium loss cause hypokalemia, which includes congenital adrenal hyperplasia, primary aldosteronism, RTA, Bartter syndrome, and Liddle syndrome. Thus, it is necessary to identify whether renal potassium loss has occurred [16]. RTA in Sjögren’s due to TIN is characterized by distal (type I) RTA, which is more common than proximal (type II) RTA (Fanconi syndrome) [17]. Pathogenesis of hypokalemia in RTA includes decreased delivery of sodium and hydrogen in the distal tubule, defective H+-ATPase activity, bicarbonate excretion, and secondary hyperaldosteronism leading to alkaline pH. Hypokalemic paralysis is usually seen three months to four years before the onset of typical symptoms of keratoconjunctivitis sicca in patients with Sjögren’s syndrome secondary to distal RTA [4]. Autoantibodies to NaCl cotransporter and carbonic anhydrase enzymes have been identified in patients with primary Sjögren’s syndrome and correlated with RTA [18].

Distal RTA occurs due to a defect in the distal nephron, which increases back-diffusion of hydrogen ions from the collecting duct lumen to the blood or inadequate transport of hydrogen ions. The defect in hydrogen ion secretion also impairs ammonia secretion from the distal tubule, which impairs the ability of nephrons to acidify the urine. Thus, the urine pH cannot be lowered normally. This results in normal anion gap metabolic acidosis with an alkaline pH of urine (greater than 5.5). It also causes hypokalemia due to potassium excretion. However, severe hypokalemia causing paralysis as an initial presentation of dRTA is a rare occurrence in secondary Sjögren’s [19].

Hypokalemic periodic paralysis, one of the inherited channelopathies, caused by mutations in the gene that regulates sodium or potassium channels, is often on the differential diagnosis as it causes episodes of weakness or paralysis secondary to low potassium levels. Other channelopathies include hyperkalemic periodic paralysis, which is caused by mutations in sodium channels, leading to paralysis that is triggered by high levels of potassium, and Andersen-Tawil syndrome, which is a rare condition caused by mutations in potassium channels, often associated with periodic paralysis, arrhythmias, and other presentations [10].

Hyperthyroidism and hypothyroidism can cause muscle weakness and episodes of paralysis, thus, it becomes essential to work up a patient to rule out these conditions, particularly in individuals who have a predisposition to periodic paralysis [10].

Chronic acidosis leads to proximal reabsorption of citrate, bone resorption, alkaline urine, and hypocitraturia, and hypercalciuria will lead to nephrolithiasis or nephrocalcinosis [18]. Patients with pSS and TIN or glomerular disease usually have a favorable diagnosis, but a high risk of chronic kidney disease is present in patients with TIN. Therefore, screening must be performed by checking the urine protein/creatinine ratio in patients with systemic pSS at least once a year to detect renal complications early [20].

Sjögren’s syndrome in patients with sicca symptoms requires secretagogues like hydroxypropyl methylcellulose and polyvinyl alcohol 0.5%. Topical cyclosporine eye drops can also be used for refractory sicca symptoms, and corneal patches and boric acid ointment can be used if corneal ulcerations are present. Propionic acid gels are used for vaginal dryness. To stimulate secretions, pilocarpine (5 mg thrice daily) and cevimeline (30 mg thrice daily) are used. Patients of Sjogren's with mild joint symptoms such as mild arthritis, arthralgia, and myalgia are treated with nonsteroidal anti-inflammatory drugs (NSAIDs) in anti-inflammatory doses given daily or as needed, depending upon symptom frequency. Those not responding to NSAIDs and with moderate to severe symptoms require DMARDs such as hydroxychloroquine or low-dose weekly methotrexate, depending upon the severity of symptoms. Patients with RTA are treated with sodium bicarbonate (0.5-2 mmol/kg in four divided doses). Patients with systemic manifestations will require additional monoclonal antibodies like rituximab and glucocorticoids [15]. Although corticosteroids are the preferred treatment of choice in TIN, the question of their benefit in pSS associated with TIN is still unclear. Other treatment options are considered in patients who are intolerant to steroids or those with refractory disease. The use of mycophenolate mofetil resulted in a significant improvement in kidney function [1].

## Conclusions

The atypical presentation of Sjögren's syndrome with hypokalemia leads to diagnostic delay, as further investigations are not usually done in the absence of classic Sjögren's symptoms and lead to misdiagnosis of recurrent and relapsing hypokalemic periodic paralysis.

In patients presenting with hypokalemic periodic paralysis, proper evaluation should be done to determine the cause of hypokalemia. When labs suggest normal anion gap metabolic acidosis in the presence of renal potassium loss, dRTA in Sjögren's syndrome should be considered, especially in young women. Further investigation with ABG, urine analysis, serological profile for antibodies, and, if required, renal biopsy should be considered so that early diagnosis and treatment can be initiated with low-dose corticosteroid, potassium, and bicarbonate replacement to prevent life-threatening complications and death.
